# Circulating BMP9 Protects the Pulmonary Endothelium during Inflammation-induced Lung Injury in Mice

**DOI:** 10.1164/rccm.202005-1761OC

**Published:** 2021-06-01

**Authors:** Wei Li, Lu Long, Xudong Yang, Zhen Tong, Mark Southwood, Ross King, Paola Caruso, Paul D. Upton, Peiran Yang, Geoffrey A. Bocobo, Ivana Nikolic, Angelica Higuera, Richard M. Salmon, He Jiang, Katharine M. Lodge, Kim Hoenderdos, Rebecca M. Baron, Paul B. Yu, Alison M. Condliffe, Charlotte Summers, Sussan Nourshargh, Edwin R. Chilvers, Nicholas W. Morrell

**Affiliations:** ^1^ Department of Medicine, School of Clinical Medicine, University of Cambridge, Cambridge, United Kingdom; ^2^ William Harvey Research Institute, Barts and the London School of Medicine and Dentistry, Queen Mary University of London, London, United Kingdom; ^3^ Cardiovascular Medicine Division and; ^5^ Division of Pulmonary and Critical Care Medicine, Department of Medicine, Brigham and Women’s Hospital–Harvard Medical School, Harvard University, Boston, Massachusetts; ^4^ Cardiovascular Medicine Division, Department of Medicine, Massachusetts General Hospital, Boston, Massachusetts; ^6^ National Heart and Lung Institute, Imperial College London, London, United Kingdom; and; ^7^ Department of Infection, Immunity and Cardiovascular Disease, University of Sheffield, Sheffield, United Kingdom

**Keywords:** BMP9, BMP signaling in endothelial cells, pulmonary endothelium, lung injury

## Abstract

**Rationale:** Pulmonary endothelial permeability contributes to the high-permeability pulmonary edema that characterizes acute respiratory distress syndrome. Circulating BMP9 (bone morphogenetic protein 9) is emerging as an important regulator of pulmonary vascular homeostasis.

**Objectives:**To determine whether endogenous BMP9 plays a role in preserving pulmonary endothelial integrity and whether loss of endogenous BMP9 occurs during LPS challenge.

**Methods:** A BMP9-neutralizing antibody was administrated to healthy adult mice, and lung vasculature was examined. Potential mechanisms were delineated by transcript analysis in human lung endothelial cells. The impact of BMP9 administration was evaluated in a murine acute lung injury model induced by inhaled LPS. Levels of BMP9 were measured in plasma from patients with sepsis and from endotoxemic mice.

**Measurements and Main Results:** Subacute neutralization of endogenous BMP9 in mice (*N* = 12) resulted in increased lung vascular permeability (*P* = 0.022), interstitial edema (*P* = 0.0047), and neutrophil extravasation (*P* = 0.029) compared with IgG control treatment (*N* = 6). In pulmonary endothelial cells, BMP9 regulated transcriptome pathways implicated in vascular permeability and cell-membrane integrity. Augmentation of BMP9 signaling in mice (*N* = 8) prevented inhaled LPS–induced lung injury (*P* = 0.0027) and edema (*P* < 0.0001). In endotoxemic mice (*N* = 12), endogenous circulating BMP9 concentrations were markedly reduced, the causes of which include a transient reduction in hepatic BMP9 mRNA expression and increased elastase activity in plasma. In human patients with sepsis (*N* = 10), circulating concentratons of BMP9 were also markedly reduced (*P* < 0.0001).

**Conclusions:** Endogenous circulating BMP9 is a pulmonary endothelial-protective factor, downregulated during inflammation. Exogenous BMP9 offers a potential therapy to prevent increased pulmonary endothelial permeability in lung injury.

**At a Glance Commentary**

**Scientific Knowledge on the Subject**

Increased pulmonary endothelial permeability is a major factor in the development of acute respiratory distress syndrome (ARDS). Evidence is emerging that circulating BMP9 (bone morphogenetic protein 9), secreted from the liver, might protect the pulmonary endothelium from injury. For example, loss of BMP9 protein or BMP9 signaling receptor contributes to the development of pulmonary arterial hypertension. The role of endogenous BMP9 in endothelial permeability remains unclear.

**What This Study Adds to the Field**

Here, we show that subacute neutralization of endogenous BMP9 leads to lung vascular injury, including enhanced endothelial permeability and neutrophil extravasation. BMP9 concentrations in plasma were markedly reduced in the setting of inflammation in mice and humans. Conversely, exogenous supplementation of BMP9 protected the lung from LPS-induced injury. This study suggests that exogenous BMP9 could offer a novel approach to prevent increased pulmonary endothelial permeability in the setting of lung injury and ARDS.

Endothelial dysfunction, inflammation, and increased capillary permeability play central roles in the pathobiology of sepsis and acute respiratory distress syndrome (ARDS) (1). Previous studies have identified important signaling pathways and protein–protein interactions within interendothelial junctions that regulate endothelial-barrier function (2). However, such knowledge has not yet resulted in approved drugs that target the increased vascular permeability present in sepsis and ARDS (1), conditions associated with an unacceptably high mortality. Exploring new pathways that preserve endothelial integrity may hasten the discovery of novel approaches to the treatment of these conditions.

BMP9 (bone morphogenetic protein 9) is a member of the TGFβ (transforming growth factor β) family that signals selectively in endothelial cells via a receptor complex comprising the high-affinity type 1 BMP receptor ALK1 (activin receptor–like kinase 1) and the type 2 BMP receptors BMPR2 (BMP receptor type 2) or activin receptor type 2A (3–5). ALK1 is expressed almost exclusively on endothelial cells (6), and its expression is 10- to 200-fold higher in lung tissue than in other tissues, indicating a particular role for ALK1-mediated signaling in homeostasis of the pulmonary endothelium (7). We previously showed that BMP9 protects human pulmonary artery endothelial cells (hPAECs) against excessive permeability induced by TNF, LPS, or thrombin (8). Moreover, administration of recombinant BMP9 protected mice against Evans Blue (EB) extravasation in the lung after intraperitoneal LPS challenge (8). Recently, adenoviral delivery of BMP9 was shown to prevent retinal vascular permeability in diabetic mice (9). Despite such evidence suggesting that augmentation of BMP9 signaling might prevent endothelial hyperpermeability, the role and regulation of endogenous BMP9 in the maintenance of endothelial-barrier function have not been investigated.

BMP9 is synthesized predominantly in the liver (10), circulates at concentrations that constitutively activate endothelial ALK1 signaling, and comprises the majority of plasma BMP activity (11). Heterozygous deleterious mutations in the *GDF2* gene (which encodes for BMP9) have been reported in patients with pulmonary arterial hypertension (12–15) and result in reduced circulating concentrations of BMP9. Furthermore, reduced plasma BMP9 proteins are found in patients with cirrhosis and portopulmonary hypertension (16, 17). Interestingly, increased vascular permeability is a well-recognized feature of chronic cirrhosis.

Given the potential protective effect of BMP9 signaling in the pulmonary endothelium, we sought to determine whether endogenous BMP9 plays a role in protecting the pulmonary endothelium and whether inflammation regulates endogenous BMP9. We further explored the potential of exogenous BMP9 as a lung vascular-protective agent in the setting of acute lung injury (ALI). Some of these results have been previously reported in the form of abstracts (18–20) and in the form of a preprint (https://doi.org/10.1101/2020.05.12.088880).

## Methods

### Human Samples

Human plasma samples were obtained from a prospectively enrolled cohort of patients admitted to the adult medical ICU (MICU) Registry of Critical Illness and from healthy human volunteers without known cardiopulmonary disease, in accordance with the institutional review board–approved protocol at Brigham and Women’s Hospital, as described previously (21–23). Written informed consent was obtained from all participants or their appropriate surrogates.

### Animal Procedures

All procedures were performed in accordance with the Home Office Animals (Scientific Procedures) Act of 1986 and were approved under Home Office Project Licenses 80/2460 and 70/8850 (to N.W.M.) and 7007884 (to S.N.).

### Murine Endotoxemia Studies

Mice were injected intraperitoneally with 2 mg/kg of LPS or vehicle. After the length of time as specified in figure legends, mice were killed using ketamine (100 mg/kg) and xylazine (10 mg/kg). Detailed tissue harvests and measurements can be found in the online supplement. *N* = 6 for each time point in LPS-treated groups. For phosphate-buffered saline (PBS)-treated control animals, three animals were included at 0, 6, 18, and 24 hours, respectively, and pooled for the final analysis (final *N* = 12 in PBS-treated group).

### BMP9 ELISA

BMP9 ELISA was performed as described previously (13, 16).

### Anti-BMP9 Treatment in Mice

**Figure 1.**
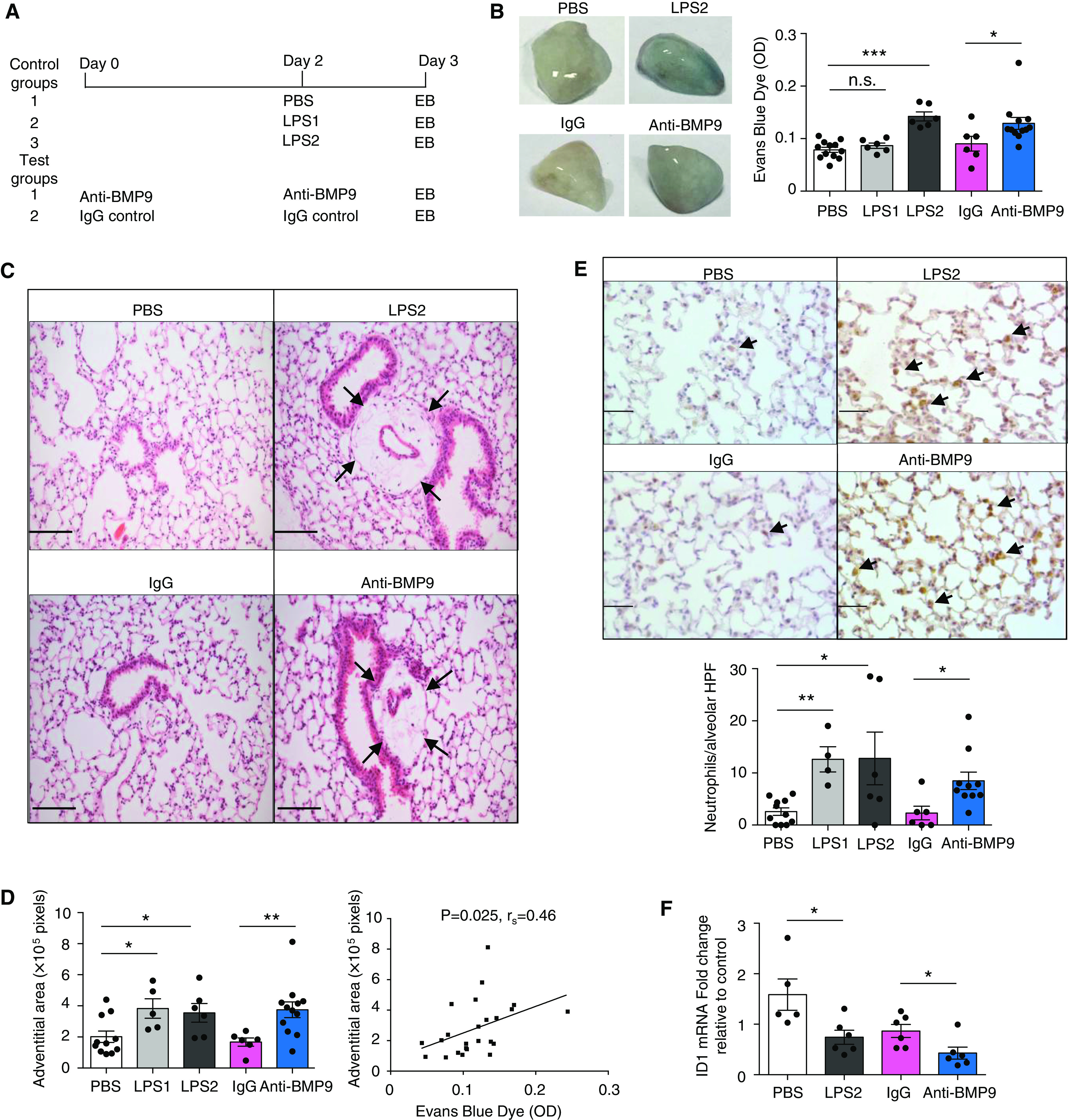
Neutralizing endogenous BMP9 (bone morphogenetic protein 9) results in lung vascular leak and neutrophil extravasation. (*A*) A schematic diagram indicating the treatment regimen. (*B*) Inhibiting endogenous BMP9 activity leads to lung vascular leak. Representative images of the lungs (left), showing the Evans Blue (EB)-stained lungs from LPS- and anti-BMP9–treated animals. Quantification of EB content in the lungs (right). *N* = 6 per group. (*C* and *D*) Anti-BMP9 treatment leads to an increase in the perivascular adventitial area, similar to LPS treatment (black arrows). (*C*) Representative pictures of hematoxylin and eosin–stained lung section. Scale bars, 100 μM. (*D*) Adventitial area in 20 random high-power fields (HPF), with its correlation to the EB content in the lung shown on the right; the Spearman correlation test was used. (*E*) Anti-BMP9 treatment increases alveolar neutrophil counts, revealed by myeloperoxidase staining. Scale bars, 50 μM. The counts were the mean of six random HPF per animal. Arrows point to neutrophils. (*F*) BMP9 activity in plasma measured by *ID1*-gene induction in human pulmonary artery endothelial cells. Serum-starved human pulmonary artery endothelial cells were treated with 1% plasma samples for 1 hour before cells were harvested for quantitative RT-PCR analysis of *ID1* gene induction. The operator was blinded to the treatment samples. For all panels, data are shown as means ± SEMs. Two-tailed Mann-Whitney tests were used to compare LPS treatment with PBS treatment and anti-BMP9 treatment with IgG treatment. *P < 0.05, **P < 0.01, and ***P < 0.001. LPS1 = LPS at 1.5 mg/kg; LPS2 = LPS at 3 mg/kg; n.s. < not significant; OD < optical density of absorbance measurements; PBS = phosphate-buffered saline.

Mice were injected intraperitoneally with 5 mg/kg of anti-BMP9 antibody (*N* = 12) or murine IgG2B isotype control (*N* = 6) on Day 0 and Day 2, and lung vascular permeability was measured on Day 3 using the EB dye-extravasation assay as described previously (8) (Figure 1A). Separate groups of control mice were injected intraperitoneally with LPS at 1.5 mg/kg, LPS at 3 mg/kg (both *N* = 6), or PBS control (*N* = 12) on Day 2, and permeability was measured on Day 3. Half of the lung tissues were harvested for measuring vascular permeability, and the other half were inflated and fixed in formalin and processed into paraffin wax blocks for histological analysis.

### Murine Inhaled-LPS Model

Mice were injected intraperitoneally with either PBS or BMP9 (at 1.5 μg/kg, *N* = 8 per group) 1 hour before being challenged with 20 μg/mouse of LPS in PBS via the intranasal route. After 24 hours, one lung was harvested for quantification of EB dye extravasation. The other lung was saved for histological analysis and for RNA extraction and quantitative reverse transcription–PCR (RT-PCR) analysis.

### Statistical Analysis

All *in vitro* experiments were conducted at least three times, and representative images are shown. Data analysis was performed using GraphPad Prism 6 (GraphPad Software). Results are shown as means ± SEMs. Statistical significance was analyzed by two-tailed nonparametric tests or one-way ANOVA as indicated in figure legends. Values of *P* < 0.05 were considered significant.

Expanded materials and methods can be found in the online supplement. Microarray data have been deposited to Gene Expression Omnibus, with the accession number GSE118353.

## Results

### Inhibition of Endogenous BMP9 Increases Lung Vascular Permeability and Neutrophil Extravasation

After the protocol shown in Figure 1A, mice treated with anti-BMP9 antibody exhibited significantly higher amounts of EB dye in their lungs than mice treated with control IgG (Figure 1B), although EB leak was only observed in the higher-LPS-dose group (Figure 1B, LPS at 3 mg/kg). Histological analysis of lung tissue demonstrated marked perivascular edema in mice exposed to either anti-BMP9 or LPS (Figure 1C, black arrows). Morphometry of arteries associated with terminal bronchioles confirmed acellular expansion of the adventitia, which correlated with the magnitude of EB dye accumulation in the lung (Figure 1D). Unexpectedly, anti-BMP9–treated mice showed a significant accumulation of neutrophils in the alveolar space, similar to that observed in LPS-treated animals (Figure 1E). To confirm that the anti-BMP9 antibody had inhibited circulating BMP9 activity, we tested plasma BMP activity by monitoring its ability to induce *ID1* gene expression in hPAECs (10, 24, 25). Indeed, plasma from anti-BMP9–treated animals showed reduced BMP activity, as evidenced by significantly lower *ID1* mRNA induction than was shown in plasma from IgG-treated control animals (Figure 1F).

To evaluate whether the effect of anti-BMP9 is specific to the pulmonary vasculature, we performed intravital microscopic imaging of the mouse cremaster microvessels to directly visualize the effects of neutralizing endogenous BMP9 under physiological conditions. Within 5 minutes of administrating anti-BMP9 antibody via the tail vein, we observed a marked increase in the extravasation of tetramethylrhodamine isothiocyanate–dextran from postcapillary venules of the cremaster muscle compared with the IgG control group (*see* Figures E1A–E1C in the online supplement). The rapidity and magnitude of this response were similar to that induced by histamine (Figure E1D). This confirms that loss of endogenous of BMP9 leads to excess vascular leak, and this effect is not limited to the pulmonary circulation.

### BMP9 Signaling Regulates Key Pathways Involved in Endothelial Cell–Membrane Integrity and Permeability

**Figure 2.**
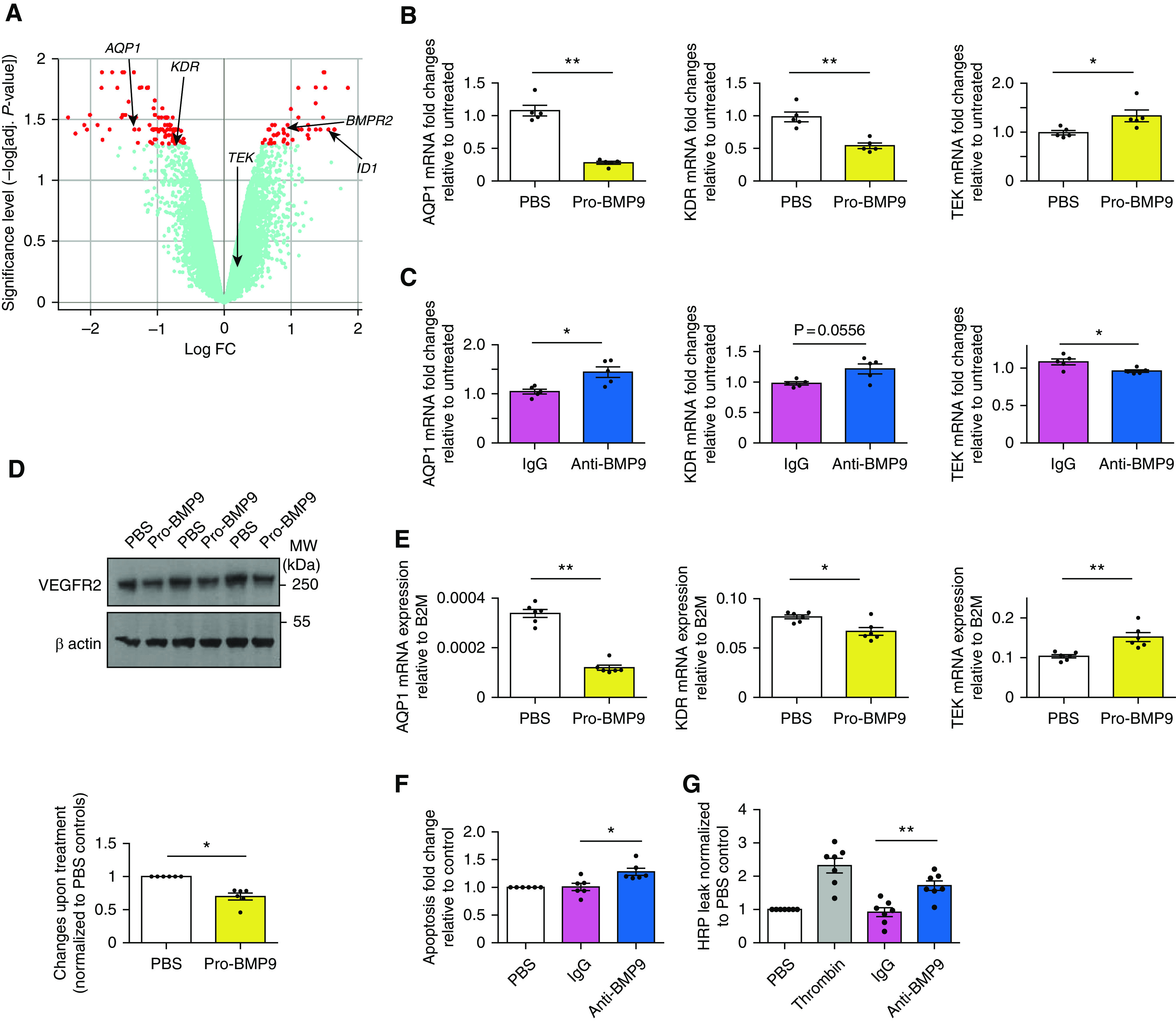
BMP9 (bone morphogenetic protein 9) signaling regulates genes involved in endothelial-cell integrity. (*A*) A volcano plot of microarray transcriptional analysis of BMP9-regulated genes. Serum-starved human pulmonary artery endothelial cells (hPAECs) were treated with 0.4 ng/ml (GF-domain concentration) pro-BMP9 (prodomain-bound form of BMP9) for 5 hours before cells were harvested for microarray analysis using the Human Gene 2.1 ST array (Affymetrix). Four independent hPAEC lines were used. Data were processed using package Oligo in R (R Foundation for Statistical Computing) (55) and normalized using robust multichip analysis (56), and comparisons were performed using the limma package (57). Resulting *P* values were corrected for multiple testing using the false discovery rate (58). Hits with adjusted. *P* values of less than 0.05 are shown in red, and those with adjusted *P* values not reaching statistical significance shown in light blue. (*B*) Validation of microarray results using quantitative RT-PCR. BMP9 signaling regulates mRNA expression of *AQP1* (aquaporin-1), *KDR* (VEGFR2), and *TEK* (Tie2) in hPAECs. *N* = 5. (*C*) Changes of gene expression in hPAECs after inhibition of BMP9 activity in fetal bovine serum with a neutralizing anti-BMP9 antibody. hPAECs were grown in endothelial basal medium with 2% fetal bovine serum and treated with IgG control or anti-BMP9 antibody (both at 20 μg/ml) for 3 hours (for *TEK*) or 5 hours (for *AQP1* and *KDR*) before cells were harvested for RNA extraction and quantitative RT-PCR analysis. *N* = 5. (*D*) BMP9 treatment suppresses VEGFR2 total proteins. Serum-starved hPAECs were treated with pro-BMP9 (0.4 ng/ml GF-domain concentration) for 5 hours (*N* = 6). Three independent treatments were run on the same Western blot and are shown. Quantification was performed using ImageJ (Wayne Rasband, National Institutes of Health), and loading was corrected by β actin controls. Changes upon BMP9 treatment relative to PBS controls were calculated and shown as means ± SEMs. A two-tailed Wilcoxon test was used. (*E*) BMP9 regulates *AQP1*, *KDR* and *TEK* expressions in human pulmonary microvascular endothelial cells (hPMECs). (*F*) In hPMECs, anti-BMP9 treatment leads to enhanced apoptosis measured using Caspase 3/7 Glo assay. (*G*) Anti-BMP9 treatment in hPMECs causes enhanced monolayer permeability measured by HRP–Transwell assay as described previously (8). For *B*, *C*, *E*, *F*, and *G*, means ± SEMs are shown, and two-tailed Mann-Whitney tests were used. *P < 0.05 and **P < 0.01. adj. = adjusted; B2M = β_2_-microglobulin; BMPR2 = BMP receptor type 2; FC = fold change; GF = growth factor; HRP = horseradish peroxidase; MW = molecular weight; PBS = phosphate-buffered saline.

To elucidate potential mechanisms by which BMP9 might act as an endothelial-protective factor, we performed a microarray analysis of global differential gene expression in hPAECs in response to BMP9. We used pro-BMP9 (prodomain-bound form of BMP9), which is the circulating form (11), at a concentration representative of those measured in healthy human plasma (16, 26), and exposed hPAECs for 5 hours. When using a threshold-adjusted *P* value of 0.05 as a cutoff, BMP9 upregulated the expression of 30 genes and downregulated 85 genes (Tables E1 and E2). However, the continuum changes in the adjusted *P* values for both up- and downregulated genes indicate that more transcripts are likely to be regulated than those passing this threshold (Figure 2A). Pathway analysis showed that BMP9-regulated pathways include TGFβ signaling, cytokine–cytokine receptor interaction, and Rap1 signaling (Tables E3 and E4). Cellular-component gene-ontology analysis revealed that BMP9-regulated genes are highly enriched in the plasma membrane and extracellular space (Figure E2 and Table E5). As expected, BMP9 increased the expression of *BMPR2* and *ID1* (3) (Figure 2A). Of the genes known to regulate endothelial permeability, pro-BMP9 treatment downregulated *AQP1* (encoding aquaporin-1) and *KDR* (encoding VEGFR2) and upregulated *TEK* (encoding Tie2) (Figure 2A), which were further validated by quantitative RT-PCR analysis (Figure 2B).

Next, we performed the converse experiments by selectively neutralizing BMP9 in serum from fetal bovine serum–containing endothelial growth media. Reciprocal changes in gene expression (i.e., a significant increase in *AQP1* and *KDR* expression and a reduction in *TEK* expression) were obtained (Figure 2C), supporting that endogenous serum BMP9 regulates these genes even in the presence of other serum factors. Immunoblotting confirmed that BMP9 treatment reduced VEGFR2 protein levels (Figure 2D). AQP1 expression in cultured hPAECs was too low to be robustly detected by Western blotting.

Because the lung microvascular endothelium rather than the conduit artery endothelium is involved in lung hyperpermeability, we further examined responses in human pulmonary microvascular endothelial cells (hPMECs). Pro-BMP9 signaled more potently in hPMECs (half maximal effective concentration [EC_50_] = 7.2 ± 1.5 pg/ml) than in hPAECs (EC_50_ = 81 ± 23 pg/ml, Figure E3A) and robustly induced known BMP9 target genes such as *ID1* and *BMPR2* (Figure E3B). BMP9 treatment also suppressed *AQP1* and *KDR* expression, and induced *TEK* expression in hPMECs (Figure 2E). Functionally, anti-BMP9 treatment in hPMECs led to enhanced apoptosis (Figure 2F) and increased permeability (Figure 2G) compared with IgG treatment. Knockdown of *BMPR2* by siRNA reversed the protection by BMP9 from LPS-induced leak in the h PMEC monolayer (Figure E3C). Consistent with these observations, we also observed loss of VE-cadherin junctions when hPAECs were treated with anti-BMP9 antibody (Figure E4). Together, these data strongly support a role of BMP9 signaling in protecting endothelial cell–membrane integrity and barrier function.

### Exogenous BMP9 Protects Mice from ALI in Response to Inhaled LPS

**Figure 3.**
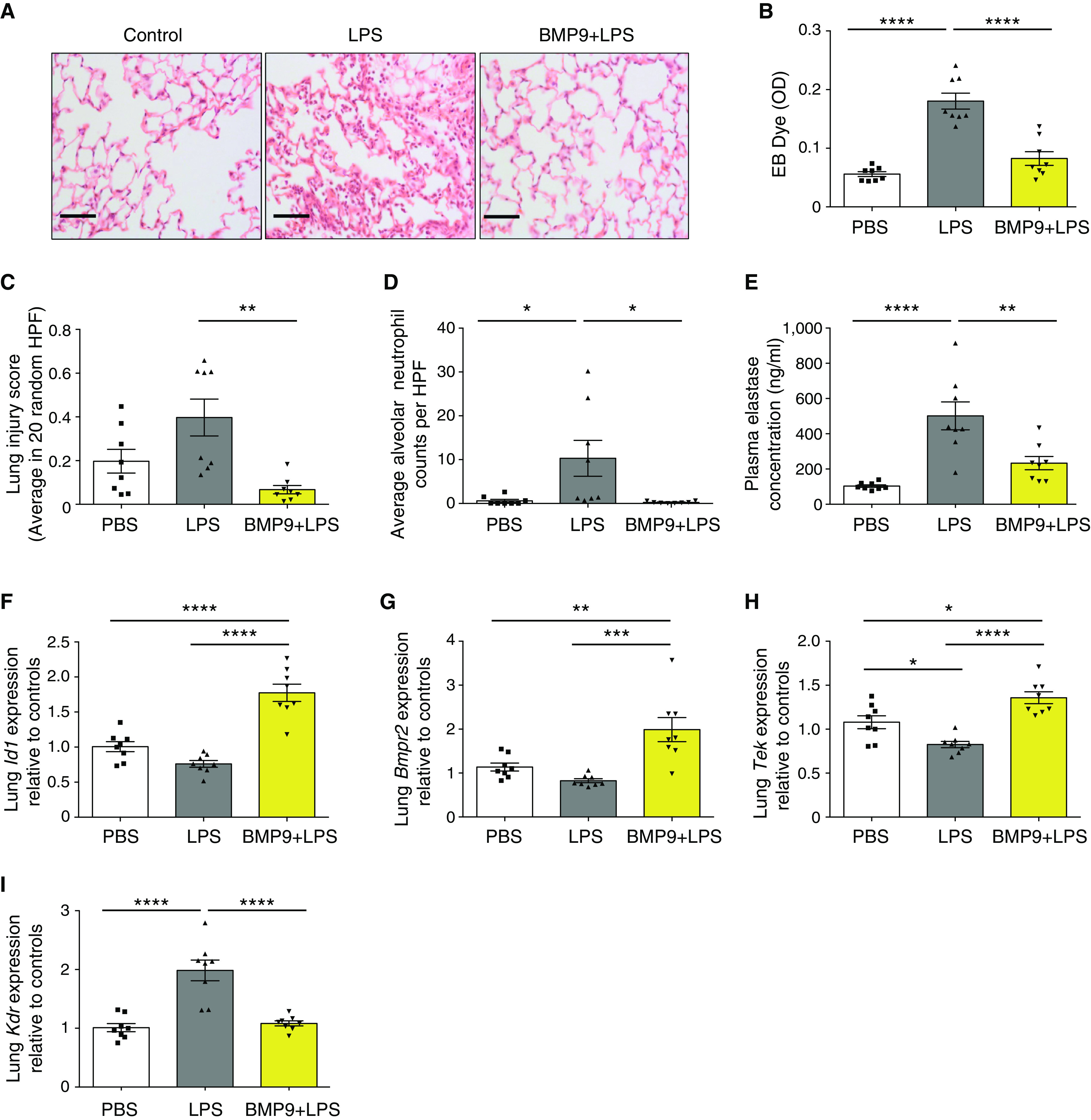
BMP9 (bone morphogenetic protein 9) prevents vascular leak and lung injury in inhaled LPS–challenged mice and involves *TEK* (Tie2) and *KDR *(VEGFR2). (*A*) Representative images of hematoxylin and eosin–stained lung tissues. Mice were challenged intranasally with LPS (at 20 μg/mouse) for 24 hours before lungs were harvested for immunohistological examination. Scale bars, 100 μM. (*B*) BMP9 prevented vascular leak measured by EB dye retained in the lungs. (*C*) BMP9 protected acute lung injury induced by inhaled LPS. Lung injury scores were based on 20 HPF per animal as per protocol from the American Thoracic Society Workshop report (27) (more details can be found in the Methods in the online supplement). (*D*) Administration of BMP9 prevented the extravasation of neutrophils into the alveolar space. Neutrophils were counted from the hematoxylin and eosin–stained slides on the basis of the shape of the cells and nuclei. (*E*) Administration of BMP9 prevented the increase of plasma elastase after inhaled-LPS challenge. (*F*–*I*) Lung mRNA expression measured by quantitative RT-PCR. *RPL32* was used as the housekeeping gene. The operator was blinded to the treatment in this experiment. For all panels, means ± SEMs are shown; one-way ANOVA and Tukey’s *post hoc* test were used. *P < 0.05, **P < 0.01, ***P < 0.001, and ****P < 0.0001. *Bmpr2* = BMP receptor type 2; EB = Evans Blue; HPF = high-power fields; OD = optical density of absorbance measurements; PBS = phosphate-buffered saline.

Acute inhalation of LPS initiates epithelial-cell damage and causes lung vascular hyperpermeability and injury. To further explore the potential therapeutic value of BMP9 (8), we questioned whether administration of exogenous BMP9 prevents ALI in such a murine model. As expected, inhalation of LPS led to pulmonary inflammation and congestion (Figure 3A) and increased lung vascular permeability measured by extravasation of EB dye (Figure 3B). All of these features were completely prevented by pretreating mice with BMP9. The degree of lung injury was scored following the recommendation of the American Thoracic Society workshop report (27) (Figure 3C), and the number of neutrophils extravasated into alveoli were counted (Figure 3D). Supplementation of BMP9 resulted in a complete protection against LPS-induced lung injury and neutrophil extravasation. Interestingly, in this model, plasma concentrations of NE (neutrophil elastase) were also elevated after LPS treatment, and this increase was prevented in the BMP9-pretreated animals (Figure 3E), suggesting an antiinflammatory role of BMP9. To confirm target engagement, we measured the changes in BMP9-regulated genes in the lung. As expected, administration of BMP9 led to an enhancement in BMP signaling, as evidenced by the elevated mRNA expression of the BMP9 target genes *Id1* and *Bmpr2* (Figures 3F and 3G). Of note, consistent with the results from *in vitro* studies (Figure 2), the expression of *Tek* was significantly reduced after LPS challenge and was restored by BMP9 treatment (Figure 3H). Furthermore, LPS treatment led to a twofold increase in the expression of *Kdr*, which was completely prevented by pretreatment with BMP9 (Figure 3I).

### Plasma BMP9 Is Suppressed during Endotoxemia

**Figure 4.**
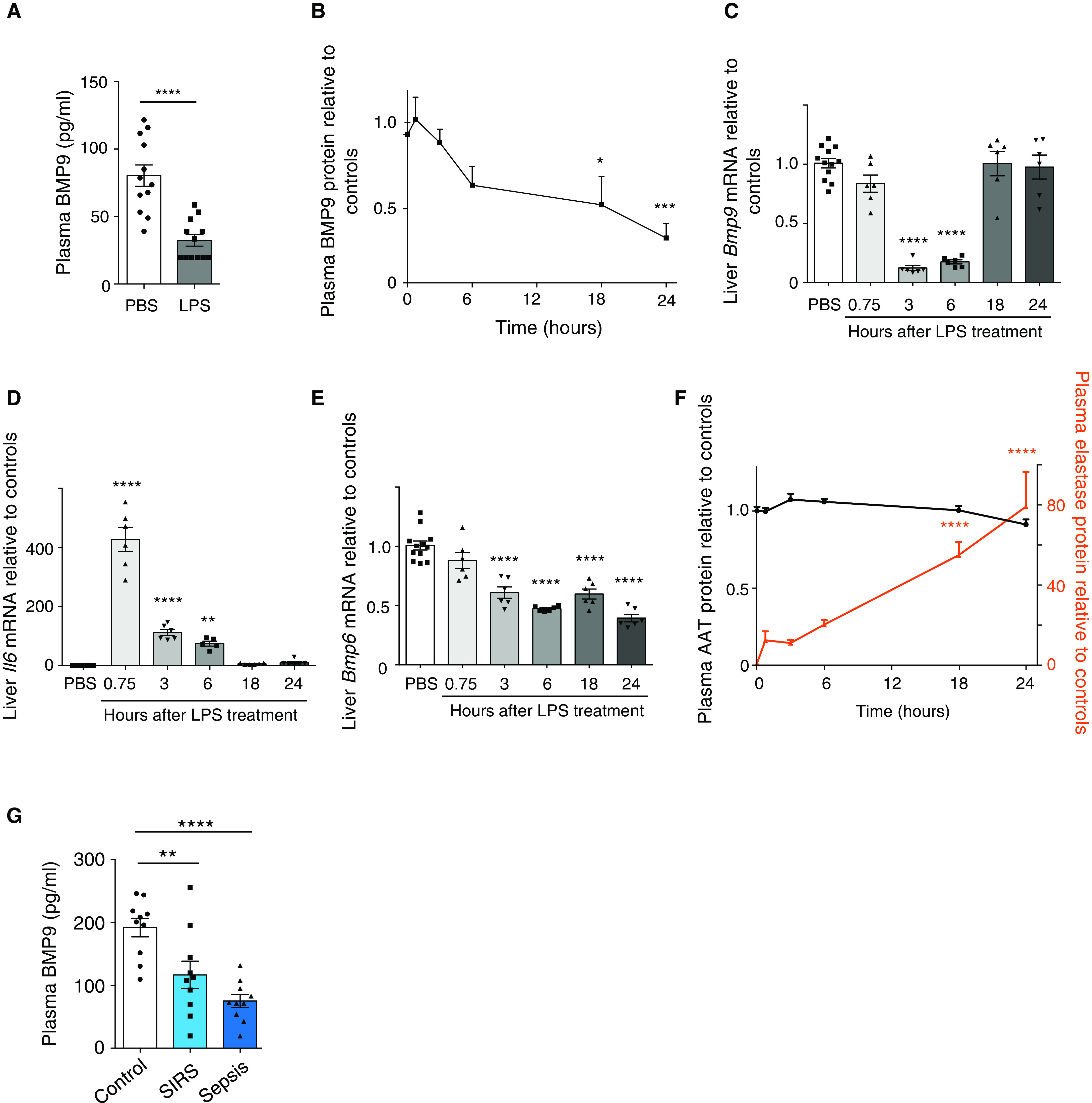
Endogenous BMP9 (bone morphogenetic protein 9) is reduced in endotoxemic mice and patients with sepsis. (*A*) Circulating BMP9 concentrations are significantly reduced in a murine endotoxemia model. Mice were treated with 2 mg/kg of LPS intraperitoneally for 18 hours before plasma samples were taken for BMP9 measurement (*N* = 12). Data are shown as means ± SEMs. A two-tailed unpaired *t* test was used. (*B*) Dynamic changes in circulating BMP9 after LPS-induced inflammation. Mice were treated with 2 mg/kg of LPS intraperitoneally and killed at 0, 0.75, 3, 6, 18, and 24 hours (*N* = 6 per group). Three animals were treated with PBS at each time point and used as control animals. Concentrations of BMP9 in plasma were measured by ELISA, normalized to controls. (*C*–*E*) Dynamic changes of liver mRNA expression relative to controls after LPS challenge. Data were analyzed using the ΔΔCt method, using *RPL32* as the housekeeping gene. (*F*) Changes in plasma elastase (red line) and AAT (alpha-1 antitrypsin; black line) protein concentrations relative to controls during endotoxemia. The actual control value for AAT is 3.44 ± 0.09 mg/ml, and the actual control value for elastase is 128.6 ± 26.8 ng/ml. (*G*) Plasma BMP9 concentrations from patients with SIRS or sepsis are significantly lower than those from healthy control subjects. In measurements for *B*–*G*, means ± SEMs are shown, and one-way ANOVA and the Dunnett *post hoc* test against controls were used. *P < 0.05, **P < 0.01, ***P < 0.001, and ****P < 0.0001. PBS = phosphate-buffered saline; SIRS = systemic inflammatory response syndrome.

Given that both depletion and supplementation of BMP9 impact lung vascular permeability, we questioned whether circulating BMP9 protein is reduced *per se* in the setting of systemic inflammation in humans and mice. Eighteen hours after intraperitoneal LPS administration, mice exhibited a systemic inflammatory response, as evidenced by a reduction in platelet numbers and an increase in alveolar neutrophil counts (Figures E5A and E5B). Concentrations of NE were significantly elevated in both plasma and BAL fluid (Figures E5C and E5D). As hypothesized, circulating concentrations of BMP9 were markedly decreased in this murine model (Figure 4A).

To further delineate changes in the endogenous BMP9 during inflammation, we performed a time-course study to track the mRNA and protein changes in BMP9 after the onset of endotoxemia in mice. Plasma BMP9 concentrations decreased from 6 hours after LPS exposure and continued to fall at the 24-hour time point (Figure 4B). Hepatic *Bmp9* mRNA were suppressed by around 80% at 3 hours after LPS exposure (Figure 4C) but returned to control values by 18 hours, despite the continued reduction in plasma BMP9 protein concentrations at these time points. As comparators, we measured liver mRNA for IL-6 to monitor the inflammatory response, and BMP6, another BMP known to be expressed in the liver (28). There was a sharp increase in *Il6* mRNA at 45 minutes after LPS administration, which fell at 3 hours and returned to baseline at 18 hours (Figure 4D). *Bmp6* mRNA was reduced to ∼60% of that in the control animals by 3 hours and remained suppressed throughout the 24-hour period (Figure 4E). These comparators confirm that the transient reduction of *Bmp9* mRNA is unique to BMP9 and is not due to the global suppression and recovery of mRNA synthesis in the liver.

Because the continued reduction in plasma BMP9 during mouse endotoxemia could not be explained fully by the changes in hepatic *Bmp9* mRNA alone, we investigated whether BMP9 might also be degraded by plasma proteases. Inflammation leads to the activation of neutrophils, which release large amounts of proteases, especially elastase (29); we therefore investigated whether LPS challenge causes changes in circulating NE concentrations. Compared with PBS-treated control animals, administration of LPS caused a 10-fold increase in the plasma elastase concentration at 45 minutes and an 80-fold increase at 24 hours (Figure 4F). Because AAT (alpha-1 antitrypsin) is the major NE inhibitor in plasma (30), we also measured AAT liver mRNA and plasma protein concentrations. AAT mRNA were largely unchanged over the first 6 hours but decreased to about 50% of those in control animals at 18 and 24 hours (Figure E6A). Using an ELISA that specifically detects the native and active form of AAT (Figure E6B) (31), we observed that plasma concentrations of AAT were largely unchanged throughout the 24-hour time course after LPS challenge (Figure 4F). This indicated that the 80-fold increase in NE protein concentrations was not counteracted by a similar fold increase in this endogenous inhibitor, leading to an imbalance favoring heightened elastase activity during endotoxemia. To examine whether circulating BMP9 are reduced in patients with systemic inflammatory response syndrome (SIRS) and sepsis, plasma BMP9 concentrations were measured in 10 patients with SIRS and 10 patients with sepsis, all sampled within 72 hours of admission to the MICU, and 10 age- and sex-matched healthy control subjects. The clinical characteristics and the demographics of the subjects are summarized in Table E6 and are notable for the presence of positive microbiological culture results and an increased reliance on vasopressors for blood-pressure support in the patients with sepsis compared with the other groups. Importantly, plasma BMP9 concentrations were significantly reduced in patients with SIRS and further reduced in patients with sepsis, compared with those in healthy control subjects (Figure 4G).

### BMP9 Is a Substrate for NE

**Figure 5.**
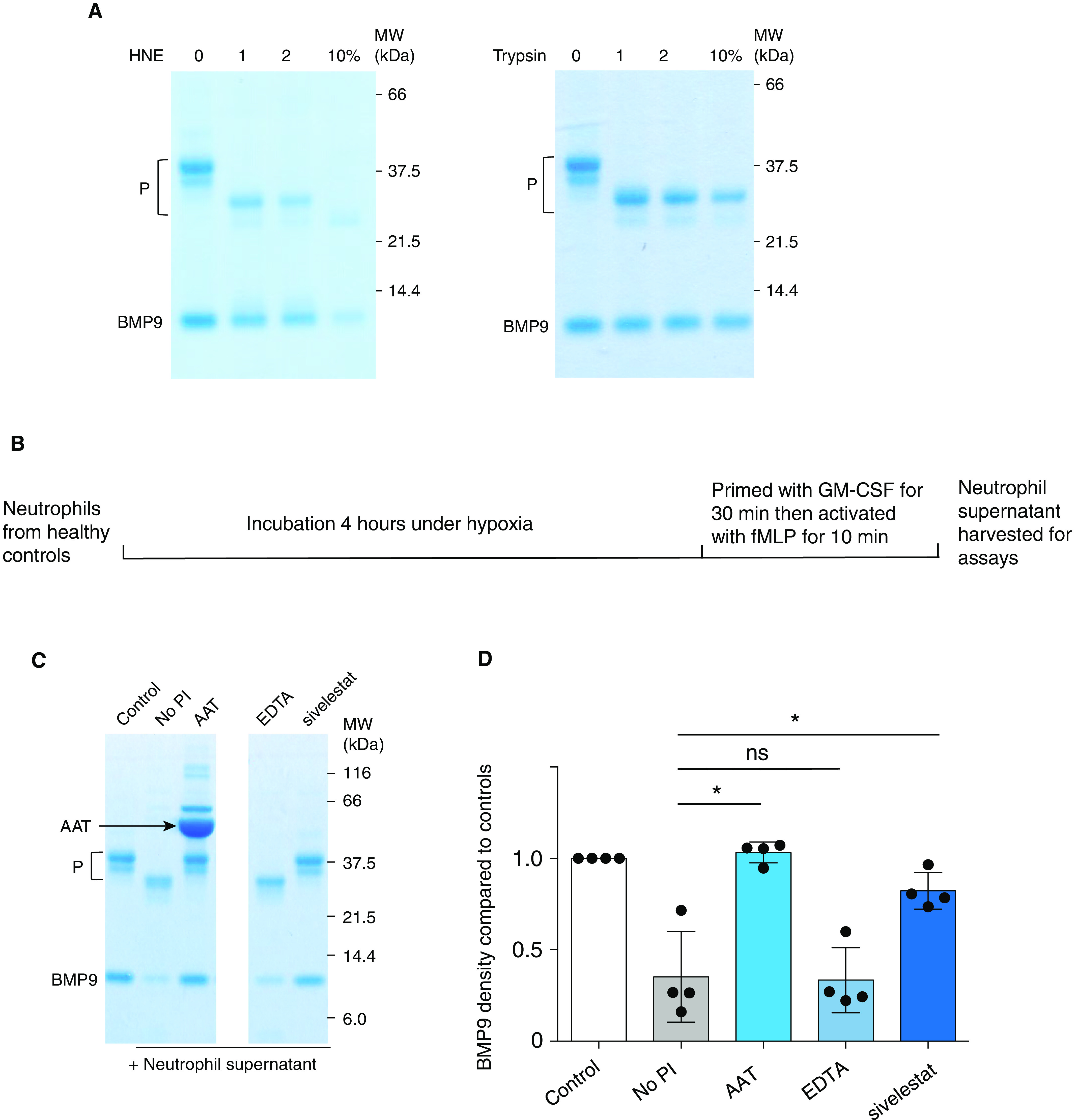
BMP9 (bone morphogenetic protein 9) is a substrate for NE (neutrophil elastase). (*A*) BMP9 is a direct substrate of elastase. Purified pro-BMP9 (prodomain-bound form of BMP9) was incubated with recombinant HNE (human NE) or trypsin at indicated concentration (% w/w) in phosphate-buffered saline overnight, and the mixture was fractionated by sodium dodecyl sulfate–polyacrylamide gel electrophoresis under reducing conditions and visualized by Coomassie Blue staining. (*B*) A schematic diagram illustrating the generation of supernatants from activated neutrophils. Neutrophils were isolated from the peripheral blood of healthy volunteers and incubated under hypoxia for 4 hours before priming with GM-CSF and activation with fMLP as described previously (29). (*C* and *D*) NE is the major protease cleaving pro-BMP9 in the activated neutrophil supernatant. Pro-BMP9 was incubated with the supernatant from activated neutrophils in the presence or absence of a panel of PI overnight, and the mixture was fractionated by sodium dodecyl sulfate–polyacrylamide gel electrophoresis under reducing conditions and visualized by Coomassie Blue staining. A representative gel from four independent experiments is shown in *C* (two parts of the same gels are shown), and the quantification of BMP9 bands from four experiments are shown in *D*. Means ± SEMs are shown, and a two-tailed, Mann-Whitney test was used. *P < 0.05. AAT = alpha-1 antitrypsin; EDTA = ethylenediaminetetraacetic acid; fMLP = formylmethionylleucylphenylalanine; MW = molecular weight; ns = not significant; P = prodomain; PI = protease inhibitors.

Finally, we sought to confirm whether BMP9 can be cleaved by NE. Using purified recombinant proteins, pro-BMP9 can be cleaved efficiently by NE, despite being highly resistant to trypsin digestion (Figure 5A). Next, we questioned whether NE in plasma could contribute to BMP9 cleavage, as primed circulating neutrophils (with increased capacity for systemic degranulation) were identified in patients with ARDS (32), activated neutrophils release a number of proteases on degranulation (29), and significantly higher concentrations of NE were found in plasma from endotoxemic mice (Figure 4F). Purified human peripheral-blood neutrophils were activated *in vitro* to degranulate and release proteases into the culture supernatant as described previously (29) (Figure 5B). Pro-BMP9 was incubated with supernatants derived from activated neutrophils, in the presence or absence of a panel of protease inhibitors. Activated neutrophil supernatants cleaved BMP9 effectively, and this process was blocked by AAT and sivelestat, a selective NE inhibitor (Figures 5C and 5D), but was not blocked by the chelating agent ethylenediaminetetraacetic acid, suggesting that metalloproteases do not play a role. Taken together, these findings suggest that NE contributes to BMP9 cleavage in the setting of inflammation.

## Discussion

The present study provides evidence that endogenous BMP9 is an important protective factor for the pulmonary vascular endothelium that is downregulated during inflammation. Selective inhibition of circulating BMP9 induced heightened lung vascular leak. Such a finding is consistent with reports that ALK1-Fc, a ligand trap of BMP9, causes peripheral edema as a common side effect in clinical trials (33, 34). In addition, loss of the major type 2 receptor for BMP9, BMPR-2, promotes endothelial permeability and contributes to the development of pulmonary arterial hypertension (8, 35). Because ALK1 is particularly highly expressed on the lung vascular endothelium (7), we speculate that the pulmonary circulation is particularly dependent on the ALK1-mediated BMP9 signaling to maintain barrier function.

Emerging roles of BMP signaling in vascular biology, particularly in endothelial cells, have been recognized and reviewed recently (36). There may be important context-specific differences in the roles of specific BMPs in the regulation of endothelial-barrier function. For example, there is evidence that specific BMPs, including BMP2, BMP4, and BMP6 (37–39), destabilize endothelial-cell junctions to increase vascular permeability. On the other hand, recombinant BMP2 and BMP4 have also been shown to play a protective role in endotoxin-induced ALI (40, 41). BMP 2, 4, and 6 must act through mechanisms different from those of BMP9 because only BMP9 and BMP10 signal specifically through the ALK1-mediated pathway in vascular endothelial cells.

The circulating form of BMP9 at physiological concentrations regulates the transcription of gene sets highly associated with the plasma membrane and extracellular space. This is consistent with previous findings either using BMP9-treated human dermal microvascular endothelial cells (42) or comparing wild-type with *Bmpr2*^-knockout^ endothelial cells (43). Among the BMP9-regulated genes are three receptors controlling critical pathways involved in endothelial permeability. Importantly, we show that *in vivo*, the protection by BMP9 against lung vascular leak in a murine ALI model was associated with the preservation of *TEK* and *KDR* expression in the LPS-exposed lungs. The effect of VEGF signaling via VEGFR2 to induce vascular leak has been extensively studied (44). Consistent with a role for Tie2, increased expression of angiopoietin 2 was found in the retina of neonatal mice receiving anti-BMP9 and anti-BMP10 antibodies (45). AQP1 regulates osmotically driven water transport across microvessels in adult lungs and facilitates hydrostatically driven lung edema (46). Decreased pulmonary vascular permeability has been described in *AQP1*-null humans (47), and *AQP1* expression is increased in the capillary endothelium of alveoli from patients with ARDS (48).

It is interesting to note the inverse correlation of circulating BMP9 and elastase concentrations in the onset of endotoxemia in mice and to note that BMP9 is a direct substrate of NE *in vitro* despite its being highly resistant to trypsin digestion. Further experiments are needed to show BMP9 cleavage by NE *in vivo*. This could be challenging because we show here that NE is a major protease but not the only serine protease released by neutrophils that has the ability to cleave BMP9 (Figures 5C and 5D); therefore, elastase inhibition alone may not be enough to rescue BMP9 in the circulation. A direct detection of elastase-cleaved BMP9 fragments *in vivo* would be more informative; however, this is difficult because of the presence of very low concentrations of BMP9 in the circulation (200–400 pg/ml).

LPS challenge causing a temporary downregulation in BMP9 mRNA in the liver is consistent with a previous report that BMP9 expression is transiently reduced in three models of acute liver damage (49). Interestingly, in another study examining the changes of BMP signaling pathways after acute LPS challenge, a similar transient downregulation of Smad1/5 phosphorylation, Id1 proteins, and Bmp4 mRNA was observed in the lung tissue (50).

We previously reported that BMP9 enhances LPS-induced leukocyte recruitment to the vascular endothelium (51). This effect was observed with higher concentrations of BMP9 that likely activate the ALK2 receptor (52). The present study used lower concentrations of BMP9, and data in this study are consistent with the results reported by Burton and colleagues (35) and Long and colleagues (8). Because BMP9 can signal through both the high-affinity receptor ALK1 and the low-affinity receptor ALK2, our overall results suggest that restoration of BMP9 to the physiological-concentration range will promote BMP9 to signal through the ALK1-mediated pathway and exert beneficial antiinflammatory and endothelial-protective effects.

Microvascular leak has now been recognized as a major contributor to septic shock and is associated with increased morbidity and mortality; as yet, there is no pharmacological drug available that targets this process (53). Restoration of endothelial integrity has been shown to increase survival in three different animal models of systemic inflammation (54). Our study identifies a new and unexpected essential role for endogenous BMP9 in the maintenance of endothelial-barrier function under physiological conditions and demonstrates that circulating BMP9 are reduced in patients with sepsis and in a murine endotoxemia model. Importantly, administration of BMP9 protects against lung vascular leak in a murine ALI model. Taken together, these findings support the exploration of BMP9 as a biomarker as well as a potential therapy for the prevention of vascular permeability and lung injury associated with sepsis and ARDS.

## References

[1] Millar FR Summers C Griffiths MJ Toshner MR Proudfoot AG The pulmonary endothelium in acute respiratory distress syndrome: insights and therapeutic opportunities *Thorax* 2016 71 462 473 26968969 10.1136/thoraxjnl-2015-207461

[2] Komarova YA Kruse K Mehta D Malik AB Protein interactions at endothelial junctions and signaling mechanisms regulating endothelial permeability *Circ Res* 2017 120 179 206 28057793 10.1161/CIRCRESAHA.116.306534 PMC5225667

[3] David L Mallet C Mazerbourg S Feige JJ Bailly S Identification of BMP9 and BMP10 as functional activators of the orphan activin receptor-like kinase 1 (ALK1) in endothelial cells *Blood* 2007 109 1953 1961 17068149 10.1182/blood-2006-07-034124

[4] Scharpfenecker M van Dinther M Liu Z van Bezooijen RL Zhao Q Pukac L *et al* BMP-9 signals via ALK1 and inhibits bFGF-induced endothelial cell proliferation and VEGF-stimulated angiogenesis *J Cell Sci* 2007 120 964 972 17311849 10.1242/jcs.002949

[5] Upton PD Davies RJ Trembath RC Morrell NW Bone morphogenetic protein (BMP) and activin type II receptors balance BMP9 signals mediated by activin receptor-like kinase-1 in human pulmonary artery endothelial cells *J Biol Chem* 2009 284 15794 15804 19366699 10.1074/jbc.M109.002881 PMC2708876

[6] Seki T Yun J Oh SP Arterial endothelium-specific activin receptor-like kinase 1 expression suggests its role in arterialization and vascular remodeling *Circ Res* 2003 93 682 689 12970115 10.1161/01.RES.0000095246.40391.3B

[7] Panchenko MP Williams MC Brody JS Yu Q Type I receptor serine-threonine kinase preferentially expressed in pulmonary blood vessels *Am J Physiol* 1996 270 L547 L558 8928814 10.1152/ajplung.1996.270.4.L547

[8] Long L Ormiston ML Yang X Southwood M Gräf S Machado RD *et al* Selective enhancement of endothelial BMPR-II with BMP9 reverses pulmonary arterial hypertension *Nat Med* 2015 21 777 785 26076038 10.1038/nm.3877 PMC4496295

[9] Akla N Viallard C Popovic N Lora Gil C Sapieha P Larrivée B BMP9 (bone morphogenetic protein-9)/Alk1 (activin-like kinase receptor type I) signaling prevents hyperglycemia-induced vascular permeability *Arterioscler Thromb Vasc Biol* 2018 38 1821 1836 29880487 10.1161/ATVBAHA.118.310733

[10] David L Mallet C Keramidas M Lamandé N Gasc JM Dupuis-Girod S *et al* Bone morphogenetic protein-9 is a circulating vascular quiescence factor *Circ Res* 2008 102 914 922 18309101 10.1161/CIRCRESAHA.107.165530 PMC2561062

[11] Bidart M Ricard N Levet S Samson M Mallet C David L *et al* BMP9 is produced by hepatocytes and circulates mainly in an active mature form complexed to its prodomain *Cell Mol Life Sci* 2012 69 313 324 21710321 10.1007/s00018-011-0751-1 PMC11114909

[12] Gräf S Haimel M Bleda M Hadinnapola C Southgate L Li W *et al* Identification of rare sequence variation underlying heritable pulmonary arterial hypertension *Nat Commun* 2018 9 1416 29650961 10.1038/s41467-018-03672-4 PMC5897357

[13] Hodgson J Swietlik EM Salmon RM Hadinnapola C Nikolic I Wharton J *et al* Characterization of *GDF2* mutations and levels of BMP9 and BMP10 in pulmonary arterial hypertension *Am J Respir Crit Care Med* 2020 201 575 585 31661308 10.1164/rccm.201906-1141OC PMC7047445

[14] Eyries M Montani D Nadaud S Girerd B Levy M Bourdin A *et al* Widening the landscape of heritable pulmonary hypertension mutations in paediatric and adult cases *Eur Respir J* 2019 53 1801371 30578383 10.1183/13993003.01371-2018

[15] Wang XJ Lian TY Jiang X Liu SF Li SQ Jiang R *et al* Germline *BMP9* mutation causes idiopathic pulmonary arterial hypertension *Eur Respir J* 2019 53 1801609 30578397 10.1183/13993003.01609-2018

[16] Nikolic I Yung LM Yang P Malhotra R Paskin-Flerlage SD Dinter T *et al* Bone morphogenetic protein 9 is a mechanistic biomarker of portopulmonary hypertension *Am J Respir Crit Care Med* 2019 199 891 902 30312106 10.1164/rccm.201807-1236OC PMC6444661

[17] Owen NE Alexander GJ Sen S Bunclark K Polwarth G Pepke-Zaba J *et al* Reduced circulating BMP10 and BMP9 and elevated endoglin are associated with disease severity, decompensation and pulmonary vascular syndromes in patients with cirrhosis *EBioMedicine* 2020 56 102794 32454407 10.1016/j.ebiom.2020.102794 PMC7248419

[18] Li W Long L Hoenderdos K Upton P Yang X Condliffe A *et al* S2 Vascular quiescence factor BMP9 is regulated by inflammation and neutrophil activation [abstract] *Thorax* 2015 70 A5

[19] Li W Long L Yang X King R Southwood M Tong Z *et al* S39 Endogenous circulating BMP9 maintains endothelial barrier function [abstract] *Thorax* 2018 73 A24

[20] Li W Long L Yang X King R Southwood M Jiang H *et al* Endogenous circulating BMP9 protects lung endothelial barrier function and is down-regulated during LPS-induced inflammation [abstract] *Am J Respir Crit Care Med* 2020 201 A2470

[21] Dolinay T Kim YS Howrylak J Hunninghake GM An CH Fredenburgh L *et al* Inflammasome-regulated cytokines are critical mediators of acute lung injury *Am J Respir Crit Care Med* 2012 185 1225 1234 22461369 10.1164/rccm.201201-0003OC PMC3373064

[22] Sun X Icli B Wara AK Belkin N He S Kobzik L *et al* MICU Registry MicroRNA-181b regulates NF-κB-mediated vascular inflammation *J Clin Invest* 2012 122 1973 1990 22622040 10.1172/JCI61495 PMC3366408

[23] Malhotra R Paskin-Flerlage S Zamanian RT Zimmerman P Schmidt JW Deng DY *et al* Circulating angiogenic modulatory factors predict survival and functional class in pulmonary arterial hypertension *Pulm Circ* 2013 3 369 380 24015338 10.4103/2045-8932.110445 PMC3757832

[24] Jiang H Salmon RM Upton PD Wei Z Lawera A Davenport AP *et al* The prodomain-bound form of bone morphogenetic protein 10 is biologically active on endothelial cells *J Biol Chem* 2016 291 2954 2966 26631724 10.1074/jbc.M115.683292 PMC4742757

[25] Salmon RM Guo J Wood JH Tong Z Beech JS Lawera A *et al* Molecular basis of ALK1-mediated signalling by BMP9/BMP10 and their prodomain-bound forms *Nat Commun* 2020 11 1621 32238803 10.1038/s41467-020-15425-3 PMC7113306

[26] van Baardewijk LJ van der Ende J Lissenberg-Thunnissen S Romijn LM Hawinkels LJ Sier CF *et al* Circulating bone morphogenetic protein levels and delayed fracture healing *Int Orthop* 2013 37 523 527 23271691 10.1007/s00264-012-1750-z PMC3580113

[27] Matute-Bello G Downey G Moore BB Groshong SD Matthay MA Slutsky AS *et al* Acute Lung Injury in Animals Study Group An official American Thoracic Society workshop report: features and measurements of experimental acute lung injury in animals *Am J Respir Cell Mol Biol* 2011 44 725 738 21531958 10.1165/rcmb.2009-0210ST PMC7328339

[28] Andriopoulos B Jr Corradini E Xia Y Faasse SA Chen S Grgurevic L *et al* BMP6 is a key endogenous regulator of hepcidin expression and iron metabolism *Nat Genet* 2009 41 482 487 19252486 10.1038/ng.335 PMC2810136

[29] Hoenderdos K Lodge KM Hirst RA Chen C Palazzo SG Emerenciana A *et al* Hypoxia upregulates neutrophil degranulation and potential for tissue injury *Thorax* 2016 71 1030 1038 27581620 10.1136/thoraxjnl-2015-207604 PMC5099189

[30] Janciauskiene SM Bals R Koczulla R Vogelmeier C Köhnlein T Welte T The discovery of α1-antitrypsin and its role in health and disease *Respir Med* 2011 105 1129 1139 21367592 10.1016/j.rmed.2011.02.002

[31] Gettins PG Olson ST Inhibitory serpins: new insights into their folding, polymerization, regulation and clearance *Biochem J* 2016 473 2273 2293 27470592 10.1042/BCJ20160014 PMC5266585

[32] Chollet-Martin S Montravers P Gibert C Elbim C Desmonts JM Fagon JY *et al* Subpopulation of hyperresponsive polymorphonuclear neutrophils in patients with adult respiratory distress syndrome: role of cytokine production *Am Rev Respir Dis* 1992 146 990 996 1416430 10.1164/ajrccm/146.4.990

[33] Jimeno A Posner MR Wirth LJ Saba NF Cohen RB Popa EC *et al* A phase 2 study of dalantercept, an activin receptor-like kinase-1 ligand trap, in patients with recurrent or metastatic squamous cell carcinoma of the head and neck *Cancer* 2016 122 3641 3649 27648727 10.1002/cncr.30317 PMC6901028

[34] Bendell JC Gordon MS Hurwitz HI Jones SF Mendelson DS Blobe GC *et al* Safety, pharmacokinetics, pharmacodynamics, and antitumor activity of dalantercept, an activin receptor-like kinase-1 ligand trap, in patients with advanced cancer *Clin Cancer Res* 2014 20 480 489 24173543 10.1158/1078-0432.CCR-13-1840

[35] Burton VJ Ciuclan LI Holmes AM Rodman DM Walker C Budd DC Bone morphogenetic protein receptor II regulates pulmonary artery endothelial cell barrier function *Blood* 2011 117 333 341 20724539 10.1182/blood-2010-05-285973

[36] Bautch VL Bone morphogenetic protein and blood vessels: new insights into endothelial cell junction regulation *Curr Opin Hematol* 2019 26 154 160 30855335 10.1097/MOH.0000000000000492

[37] Benn A Bredow C Casanova I Vukičević S Knaus P VE-cadherin facilitates BMP-induced endothelial cell permeability and signaling *J Cell Sci* 2016 129 206 218 26598555 10.1242/jcs.179960 PMC4732303

[38] Hussein KA Choksi K Akeel S Ahmad S Megyerdi S El-Sherbiny M *et al* Bone morphogenetic protein 2: a potential new player in the pathogenesis of diabetic retinopathy *Exp Eye Res* 2014 125 79 88 24910902 10.1016/j.exer.2014.05.012 PMC4122600

[39] Helbing T Wiltgen G Hornstein A Brauers EZ Arnold L Bauer A *et al* Bone morphogenetic protein-modulator BMPER regulates endothelial barrier function *Inflammation* 2017 40 442 453 27995357 10.1007/s10753-016-0490-4

[40] Li Z Wang J Wang Y Jiang H Xu X Zhang C *et al* Bone morphogenetic protein 4 inhibits liposaccharide-induced inflammation in the airway *Eur J Immunol* 2014 44 3283 3294 25142202 10.1002/eji.201344287

[41] Wang K Gong J Pei L Shan S Tan W The effect of rhBMP-2 on pulmonary arterioles remodeling in endotoxin-induced acute lung injury in rats *Clin Exp Med* 2013 13 187 192 22743650 10.1007/s10238-012-0197-2

[42] Young K Conley B Romero D Tweedie E O’Neill C Pinz I *et al* BMP9 regulates endoglin-dependent chemokine responses in endothelial cells *Blood* 2012 120 4263 4273 23018639 10.1182/blood-2012-07-440784 PMC3501721

[43] Hiepen C Jatzlau J Hildebrandt S Kampfrath B Goktas M Murgai A *et al* BMPR2 acts as a gatekeeper to protect endothelial cells from increased TGFβ responses and altered cell mechanics *PLoS Biol* 2019 17 e3000557 31826007 10.1371/journal.pbio.3000557 PMC6927666

[44] Bates DO Vascular endothelial growth factors and vascular permeability *Cardiovasc Res* 2010 87 262 271 20400620 10.1093/cvr/cvq105 PMC2895541

[45] Ruiz S Zhao H Chandakkar P Chatterjee PK Papoin J Blanc L *et al* A mouse model of hereditary hemorrhagic telangiectasia generated by transmammary-delivered immunoblocking of BMP9 and BMP10 *Sci Rep* 2016 5 37366 27874028 10.1038/srep37366 PMC5118799

[46] Bai C Fukuda N Song Y Ma T Matthay MA Verkman AS Lung fluid transport in aquaporin-1 and aquaporin-4 knockout mice *J Clin Invest* 1999 103 555 561 10021464 10.1172/JCI4138 PMC408096

[47] King LS Nielsen S Agre P Brown RH Decreased pulmonary vascular permeability in aquaporin-1-null humans *Proc Natl Acad Sci U S A* 2002 99 1059 1063 11773634 10.1073/pnas.022626499 PMC117429

[48] Lai KN Leung JC Metz CN Lai FM Bucala R Lan HY Role for macrophage migration inhibitory factor in acute respiratory distress syndrome *J Pathol* 2003 199 496 508 12635141 10.1002/path.1291

[49] Breitkopf-Heinlein K Meyer C König C Gaitantzi H Addante A Thomas M *et al* BMP-9 interferes with liver regeneration and promotes liver fibrosis *Gut* 2017 66 939 954 28336518 10.1136/gutjnl-2016-313314

[50] Talati M Mutlak H Lane KB Han W Hemnes A Mutlak O *et al* NF-κB activation exacerbates, but is not required for murine Bmpr2-related pulmonary hypertension *Diseases* 2014 2 148 167

[51] Appleby SL Mitrofan CG Crosby A Hoenderdos K Lodge K Upton PD *et al* Bone morphogenetic protein 9 enhances lipopolysaccharide-induced leukocyte recruitment to the vascular endothelium *J Immunol* 2016 197 3302 3314 27647829 10.4049/jimmunol.1601219 PMC5104271

[52] Mitrofan CG Appleby SL Nash GB Mallat Z Chilvers ER Upton PD *et al* Bone morphogenetic protein 9 (BMP9) and BMP10 enhance tumor necrosis factor-α-induced monocyte recruitment to the vascular endothelium mainly via activin receptor-like kinase 2 *J Biol Chem* 2017 292 13714 13726 28646109 10.1074/jbc.M117.778506 PMC5566526

[53] Goldenberg NM Steinberg BE Slutsky AS Lee WL Broken barriers: a new take on sepsis pathogenesis *Sci Transl Med* 2011 3 88ps25 10.1126/scitranslmed.3002011 21697528

[54] London NR Zhu W Bozza FA Smith MC Greif DM Sorensen LK *et al* Targeting Robo4-dependent Slit signaling to survive the cytokine storm in sepsis and influenza *Sci Transl Med* 2010 2 23ra19 10.1126/scitranslmed.3000678 PMC2875996 20375003

[55] Carvalho BS Irizarry RA A framework for oligonucleotide microarray preprocessing *Bioinformatics* 2010 26 2363 2367 20688976 10.1093/bioinformatics/btq431 PMC2944196

[56] Irizarry RA Hobbs B Collin F Beazer-Barclay YD Antonellis KJ Scherf U *et al* Exploration, normalization, and summaries of high density oligonucleotide array probe level data *Biostatistics* 2003 4 249 264 12925520 10.1093/biostatistics/4.2.249

[57] Ritchie ME Phipson B Wu D Hu Y Law CW Shi W *et al* Limma powers differential expression analyses for RNA-sequencing and microarray studies *Nucleic Acids Res* 2015 43 e47 25605792 10.1093/nar/gkv007 PMC4402510

[58] Benjamini Y Hochberg Y Controlling the false discovery rate: a practical and powerful approach to multiple testing *J R Stat Soc B* 1995 57 289 300

